# A replicating recombinant vesicular stomatitis virus model for dairy cattle H5N1 influenza virus glycoprotein evolution

**DOI:** 10.1128/jvi.00389-25

**Published:** 2025-06-04

**Authors:** Lindsey R. Robinson-McCarthy, Kylie E. Zirckel, Holly C. Simmons, Valerie Le Sage, Kevin R. McCarthy

**Affiliations:** 1 Center for Vaccine Research, University of Pittsburgh School of Medicine 12317 , Pittsburgh, Pennsylvania, USA; 2 Department of Microbiology and Molecular Genetics, University of Pittsburgh School of Medicine 12317 , Pittsburgh, Pennsylvania, USA; Cornell University Baker Institute for Animal Health, Ithaca, New York, USA

**Keywords:** viruses, avian influenza viruses, hemagglutinin, neuraminidase, evolution, antibodies, small-molecule inhibitors

## Abstract

A panzootic of highly pathogenic avian influenza (HPAI) H5N1 viruses from clade 2.3.4.4b has triggered a multistate outbreak in US dairy cattle and an unknown number of human infections. HPAI viruses are handled in specialized biocontainment facilities. Ethical considerations limit certain evolution experiments aimed at assessing viral resistance to potential therapeutics. We have developed a replicating recombinant vesicular stomatitis virus (rVSV) where we replaced its glycoprotein with the hemagglutinin (HA) and neuraminidase (NA) genes of a 2.3.4.4b H5N1 virus (rVSV-H5N1dc2024), which enables these experiments to be performed under standard biosafety considerations. This virus grows to high titers and encodes a fluorescent reporter to track infection. We demonstrate the utility of rVSV-H5N1dc2024 in neutralization experiments, the evaluation of antibody escape, and the characterization of resistance mutations to NA inhibitors. rVSV-H5N1dc2024 or similar viruses may accelerate efforts to develop and evaluate interventions against this emerging threat to human and animal health.

**IMPORTANCE:**

Highly pathogenic avian influenza H5 viruses have spread globally, established sustained transmission in mammals, and caused human infections. Research on these viruses is restricted to high biocontainment laboratories. We report the characterization and utility of a surrogate, replicating virus that displays the two key influenza virus glycoproteins, hemagglutinin and neuraminidase, and can be safely handled in most research laboratories. This virus is amenable to the evaluation of antiviral antibodies and small-molecule inhibitors and the evolution of viral resistance to these agents. This virus can enable a wider range of researchers to study H5 viruses of pandemic concern.

## INTRODUCTION

Beginning in 2020, a lineage of highly pathogenic avian influenza (HPAI), H5 clade 2.3.4.4b, spread beyond Southeast Asia and initiated a global panzootic on six continents (1). Unexpectedly, these viruses achieved sustained transmission in US dairy cattle (2). As of April 2025, >1,000 herds in 17 states have been afflicted (3). At least 41 documented human infections with direct ties to dairy cattle have been reported (4); however, serologic surveys suggest that the case burden is far higher (5–7). Laboratory studies of HPAI require specialized containment facilities (8, 9). Ethical considerations limit efforts to understand how these viruses may evolve in the presence of potential therapeutics. These limitations may delay efforts to develop and evaluate interventions against these viruses for humans and animals.

Replicating recombinant vesicular stomatitis viruses (rVSVs) are handled at lower biosafety levels and can be modified to express foreign glycoproteins (8, 10–19). In the absence of the VSV glycoprotein (G), cell entry is dictated by the foreign glycoprotein(s). For instance, rVSV expressing the ebolavirus glycoprotein (GP) was used to identify its cellular receptor (20). Because VSV is largely apathogenic in humans, rVSVs are often vaccine candidates, including vaccines against older (non clade 2.3.4.4b) H5 HPAI and in the approved ebolavirus Zaire vaccine (Ervebo) (14, 21–24). Unlike lentivirus or other viral pseudotype systems, rVSVs are capable of sustained replication and are amenable to evolution studies. rVSVs expressing severe acute respiratory syndrome coronavirus 2 (SARS-CoV-2) spike were used to evaluate monoclonal and serum antibody escape, enabling the identification of therapeutic candidates and future antigenic variants (25, 26).

We have generated, characterized, and demonstrated the utility of a rVSV that expresses a dairy cattle H5 clade 2.3.4.4b hemagglutinin (HA) and neuraminidase (NA) (rVSV-H5N1dc2024). This virus grows to high titers, encodes a green fluorescent protein to track infection, has a monoclonal antibody (mAb) neutralization profile that approximates a matched influenza isolate, and is inhibited by a small molecule targeting the NA catalytic site. We show that rVSV-H5N1dc2024 evolves resistance to mAbs in experimental evolution studies and is suitable for evaluating NA inhibitor resistance mutations. rVSV-H5N1dc2024 and similar viruses can accelerate response efforts to the 2.3.4.4b HPAI panzootic by expanding the number of laboratories that can work with this virus.

## RESULTS

### Recombinant VSV expressing influenza glycoproteins as a surrogate for dairy cattle HPAI

**Fig 1:**
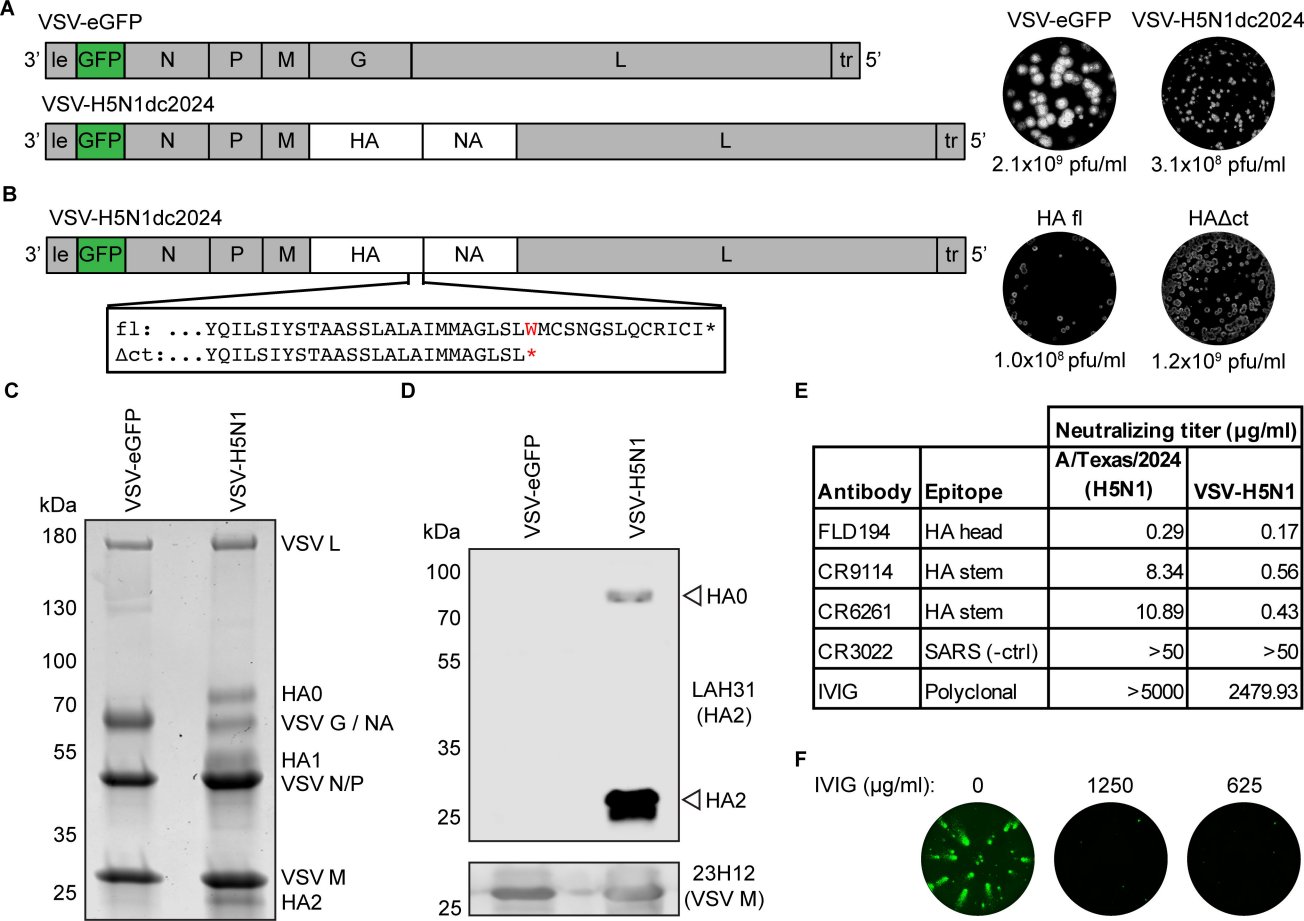
Generation of rVSV-H5N1dc2024. (**A**) Schematic of genomes, representative plaques, and infectious titers for rVSV-eGFP and rVSV-H5N1dc2024. (**B**) Identification of stop codon truncating the cytoplasmic tail of HA, representative plaques, and infectious titers of full-length (FL) and cytoplasmic tail-truncated (Δct) HA viruses. (**C**) Coomassie-stained SDS-PAGE of purified viruses. VSV G (only present in rVSV-eGFP) and NA (only in rVSV-H5N1dc2024) run at the same position, as do VSV N and VSV P. (**D**) Western blot of HA incorporation into purified virions. Unprocessed HA0 and processed HA2 are present in viral particles. (**E**) Neutralizing activity of monoclonal antibodies and IVIG against authentic A/dairy cattle/Texas/24008749001/2024 (H5N1) and rVSV-H5N1dc2024. (**F**) IVIG inhibits spread of rVSV-H5N1dc2024. eGFP-positive infected cells were imaged one day postinfection.

To generate a replication-competent virus to study the entry and evolution of highly pathogenic avian influenza virus glycoproteins at biosafety level (BSL)-2, we used the rVSV platform. We generated a molecular clone of VSV, rVSV-H5N1dc2024, in which we replaced the VSV glycoprotein with HA and NA from A/dairy cow/Texas/24–008749-001/2024, a dairy cattle-derived H5 clade 2.3.4.4b virus (Fig. 1A). The HA and NA are identical in amino acid sequence to those of the original isolate, including the polybasic cleavage site in HA. This infectious clone encodes an eGFP reporter. rVSV-H5N1dc2024 grows to titers of 3.1 × 10^8^ plaque-forming units (PFU)/mL, within one log of rVSV-eGFP encoding its own glycoprotein (Fig. 1A). When sequencing the virus, we noted a mixed population in early passages, with some sequences containing a stop codon in the HA cytoplasmic tail (Fig. 1B). We plaque-purified and grew stocks of full-length (FL) and cytoplasmic tail-truncated (Δct) viruses. The Δct virus grows to titers approximately one log higher than the FL version, similar to rVSV-eGFP.

rVSV-H5N1dc2024 efficiently incorporates both HA and NA into particles (Fig. 1C). In addition to the VSV core proteins M, N, P, and L, rVSV-H5N1dc2024 shows clear bands corresponding to HA and NA by sodium dodecyl sulfate–polyacrylamide gel electrophoresis (SDS-PAGE). HPAI HAs are processed by furin proteases in the producer cell (27, 28). To verify that the bands we observed by SDS-PAGE correspond to unprocessed HA0 and processed HA1/HA2, we performed a Western blot using antibody LAH31, which binds a conserved linear epitope in the long alpha helix of HA2 (29) (Fig. 1D). The majority of H5 incorporated into rVSV-H5N1dc2024 particles is in its processed HA1/HA2 (fusion competent) form, although some unprocessed HA0 is also present in virions.

We tested the susceptibility of rVSV-H5N1dc2024 to inhibition by H5-targeting mAbs and directly compared it to a sequence-matched authentic A/dairy cow/Texas/24–008749-001/2024 virus generated through reverse genetics (30) (Fig. 1E). In endpoint viral neutralization assays (30), rVSV-H5N1dc2024 and authentic H5N1 were inhibited by the same HA-specific mAbs and were not inhibited by SARS-CoV antibody CR3022 (31). rVSV-H5N1 showed similar sensitivity to the authentic virus to mAbs targeting the HA head, and increased sensitivity to mAbs targeting the HA stem. Intravenous immunoglobulin (IVIG), purified IgG pooled from thousands of healthy human donors (32), did not inhibit authentic H5N1 and only had very weak activity against rVSV-H5N1dc2024. Although IVIG did not efficiently prevent infection, it did prevent the spread of rVSV-H5N1dc2024 at sub-neutralizing concentrations (Fig. 1F).

### Rapid generation of antibody escape mutants

**Fig 2:**
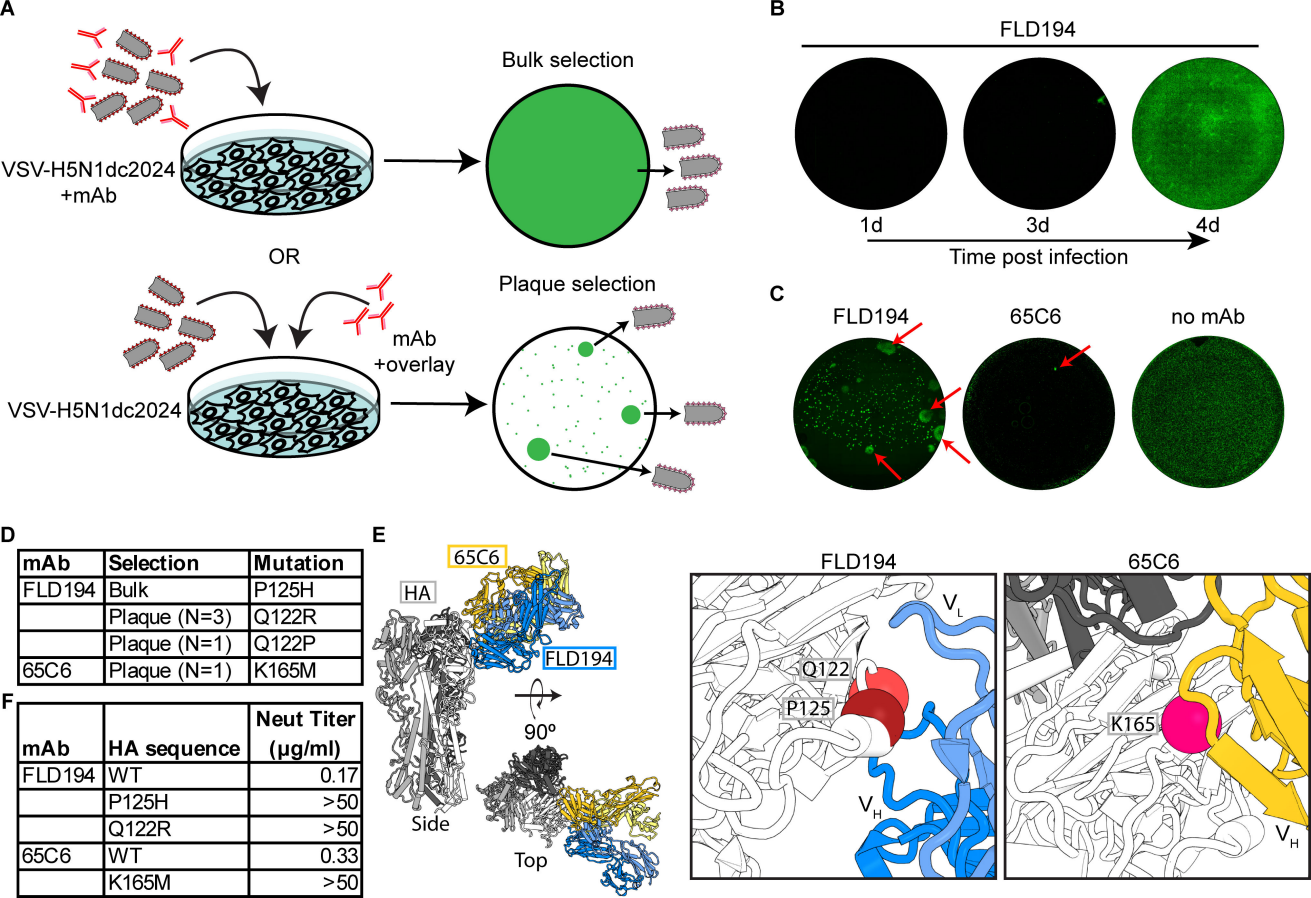
VSV-H5N1dc2024 for rapid selection of antibody escape. (**A**) Schematic of antibody escape selection strategies. (**B**) Bulk selection of resistance to FLD194 (33). Cells were imaged over time to observe viral spread by four days postinfection. (**C**) Plaque selection of resistance to FLD194 and 65C6 (34). eGFP-positive plaques were imaged three days postinfection. Arrows denote plaques that were chosen for further analysis. (**D**) Mutations identified in HA in selected viruses. (**E**) Structures of FLD194 (33) and 65C6 (35) complexed with an H5 HA (PDB: 5A3I, 9EKF). Left: superposition of FLD194 and 65C6 onto H5 HA, shown with both side and top views. Right: position of mutations identified in mAb resistant viruses. Residues identified in mutant viruses are shown as spheres: Q122 (red), and P125 (dark red) are shown in context with mAb FLD194, and K165 (magenta) is shown in context with mAb 65C6. (**F**) Neutralizing titers of mAbs with wild-type and mutant viruses.

rVSV-H5N1dc2024 is a replicating BSL-2 virus that cannot reassort with circulating influenza viruses. Given these features, we demonstrate that this virus is suitable for assessing virus escape from mAbs. Using two potently neutralizing mAbs, which engage distinct but overlapping epitopes on the HA head (33, 34), we took two approaches to generate escape mutants (Fig. 2A): first, by “bulk” selection, pre-incubating virus with mAb and growing the virus in the presence of the mAb and second, by infecting cells, then adding mAb to the plaque assay overlay, and picking large plaques that formed (26). After four days of bulk selection with mAb FLD194, we observed viral spread throughout the culture (Fig. 2B). Three days after plaque selection with both FLD194 and 65C6, we identified large plaques (four and one, respectively), which we picked and grew stocks from (Fig. 2C). We identified three separate point mutations in FLD194 selected viruses (Q122R, Q122P, and P125H) (Fig. 2D). We identified K165M in 65C6 selected virus (Fig. 2D). These mutations fall within the epitopes of the respective antibodies (Fig. 2E). All four mutant viruses were resistant to neutralization by the mAbs used in the selection experiment (Fig. 2F).

### Evaluating drug escape mutations

**Fig 3:**
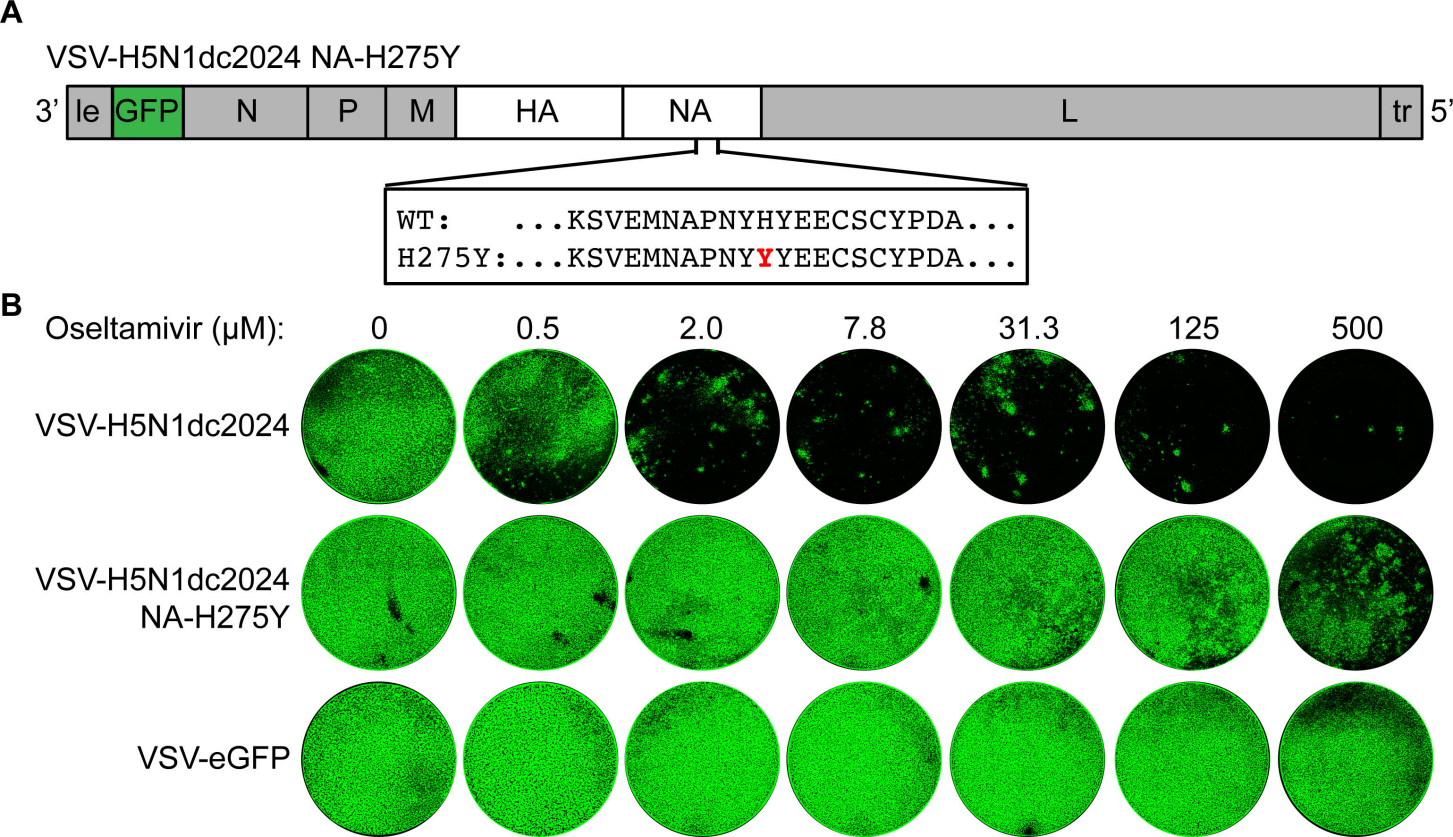
rVSV-H5N1dc2024 for assessing drug resistance. (**A**) Oseltamivir resistance mutation NA-H275Y was engineered into pVSV-H5N1dc2024-HAΔct. (**B**) Serial dilutions of oseltamivir were mixed with 100 PFU of the indicated virus. Infected cells were imaged for eGFP two days postinfection to observe viral spread.

Neuraminidase inhibitors, like oseltamivir, inhibit virus release from producer cells. Replicating viruses are therefore the best tool to determine the direct effect of inhibitors of viral spread over multiple replicative cycles. Assessing the effect of NA mutations identified by surveillance efforts by engineering these into the authentic virus requires extra scrutiny and biosafety/ethical considerations. NA H275Y confers strong resistance to oseltamivir and was recently identified in clade 2.3.4.4.b viruses infecting domesticated poultry (36, 37). To determine whether H275Y confers oseltamivir resistance in the context of clade 2.3.4.4b H5N1, we engineered this mutation into rVSV-H5N1dc2024 (Fig. 3A). Oseltamivir reduces cell-to-cell spread of rVSV-H5N1dc2024 at concentrations of 2 µM and above and does not inhibit rVSV-eGFP (Fig. 3B). rVSV-H5N1dc2024-NA-H275Y is resistant to oseltamivir, exhibiting only partial inhibition at the highest concentration tested (500 µM).

## DISCUSSION

The current outbreak of HPAI H5N1 continues to spread among dairy cattle, and the number of human cases of H5 clade 2.3.4.4b influenza virus infection continues to rise. The trajectory of this outbreak is unknown, and experiments aimed at forecasting it are limited by requirements for high-level biocontainment and gain-of-function research concerns. To facilitate a rapid response to this potential pandemic pathogen, we produced a replicating BSL-2 virus for studying H5 clade 2.3.4.4.b HPAI glycoproteins. rVSV-H5N1dc2024 incorporates HA and NA into viral particles, grows to high titers, and has a pattern of neutralization by monoclonal antibodies that approximates that of an authentic, matched influenza virus. We demonstrate that this system can be used to assess escape from monoclonal antibodies and to evaluate resistance to antiviral drugs.

In humans, antibodies to HA are the major correlate of protection from influenza virus infection (38–40). rVSV-H5N1dc2024 has HA and NA sequences matching an authentic viral isolate. It recapitulates the neutralization phenotype of the authentic virus for mAbs targeting the HA head but is more sensitive to neutralization by mAbs targeting the HA stem. Further investigation is required to understand this cause of this phenotype. While this increased neutralization sensitivity may result in overestimations of neutralization potency by stem-directed monoclonal antibodies or polyclonal serum, the difference between rVSV-H5N1dc2024 and a matched virus is approximately 10-fold. Other pseudotype systems can differ by multiple logs (41, 42). Despite its heightened neutralization sensitivity to stem antibodies, we determined that human IVIG (produced from thousands of human donors) has little-to-no neutralization activity against rVSV-H5N1dc2024. The authentic virus was not neutralized. These observations agree with recent studies showing that individual donors have serum antibodies with limited-to-no neutralizing activity against H5 clade 2.3.4.4.b viruses (30, 43). Preexisting neutralizing antibodies to this H5 are therefore likely to be of low abundance in humans. Our use of a replicating virus with a fluorescent reporter enabled us to observe inhibition of cell-to-cell spread, suggesting the presence of antibodies in the human population that may act by interfering with viral assembly or egress.

Pandemic preparedness encompasses understanding the biology of an agent, developing therapeutics and vaccines, and evaluating the consequences of viral evolution in real time. We demonstrate that rVSV-H5N1dc2024, a BSL-2 agent, is a surrogate well-suited for rapidly prioritizing experiments performed at higher biosafety levels. It is amenable to prospective studies of genetic barriers to therapeutic/prophylactic antibody resistance and to proactive assessment of known mutations that confer resistance to therapeutics. This includes assessing the effects of mutations, such as NA H275Y, which was recently identified in H5 clade 2.3.4.4b viruses in domesticated poultry (37). The virus itself is a potential vaccine candidate that can be manufactured at lower biocontainment and/or without the possibility of reassortment with circulating seasonal human viruses (14, 18, 23). The malleability of rVSVs, including H5N1dc2024, enables the rapid generation of panels of viruses with mutations to rapidly assess their significance. The eGFP reporter encoded by the virus facilitates high-throughput screening. Combined, rVSV-H5N1dc2024 and related viruses can accelerate pandemic preparedness and risk assessments.

## MATERIALS AND METHODS

### Cells

BSRT7 cells (44) and Madin-Darby canine kidney (MDCK) cells were maintained at 37°C and 5% CO_2_ in Dulbecco’s modified Eagle medium (DMEM; Thermo Fisher) or minimal essential medium (MEM; Thermo Fisher), respectively, supplemented with 10% fetal bovine serum (FBS) and 100 IU/mL penicillin/ 100 µg/mL streptomycin (pen/strep; Thermo Fisher). 293 F cells were maintained at 37°C with 8% CO_2_ in FreeStyle 293 Expression Medium (Thermo Fisher) supplemented with pen/strep.

### Plasmids

cDNA sequences corresponding to the HA and NA of A/dairy cow/Texas/24-008749-001/2024 were ordered from Integrated DNA Technologies (IDT). Coding sequences for both genes are identical to the reference sequence, with the exception of two noncoding point mutations in HA and one in NA to remove restriction sites used for cloning. To generate pVSV-H5N1dc2024, HA and NA were cloned into pVSV-eGFPΔG (10) using *Mlu*I and *Not*I restriction sites. HA and NA were separated by the VSV intergenic sequence (TTTATGAAAAAAACTAACAGCAATC) and a *Kpn*I restriction site. pVSV-H5N1dc2024-NA-H275Y was generated by site-directed mutagenesis. The pVSV-H5N1dc2024-NA-H275Y plasmid contains a stop codon in the HA cytoplasmic tail to match the sequence of rVSV-H5N1dc2024-HAΔct. The pVSV-eGFP plasmid was previously generated (45). Sequences corresponding to the heavy and light chain variable domains of antibodies FLD194, CR9114, CR6261, CR3022, 65C6, and LAH31 (29, 31, 33, 34, 46, 47) were ordered from IDT and cloned into modified pVRC8400 plasmids containing full-length human IgG1 heavy chains or human kappa or lambda light chains (48). All plasmid sequences were verified by Sanger sequencing (Azenta) or whole-plasmid nanopore sequencing (Plasmidsaurus).

### Viruses

rVSVs were generated as previously described (49), with some modifications. BSRT7 cells were infected with Fowlpox-T7 (50) and transfected with VSV genomic plasmids along with helper plasmids encoding the VSV N, P, L, and G proteins. All rVSVs were propagated at 34°C on BSRT7 cells in DMEM supplemented with 2% FBS, 25 mM 4-(2-hydroxyethyl)−1-piperazineethanesulfonic acid (HEPES), and pen/strep. Viral titers were determined by plaque assay on BSRT7 cells. Viral RNA was isolated using a QIAamp Viral RNA Mini Kit (Qiagen), and the region containing HA and NA was reverse transcribed using the Luna One-Step RT-PCR Kit (New England Biolabs). cDNA was sequenced using Oxford Nanopore Technology (Plasmidsaurus).

A/dairy cattle/Texas/24008749001/2024 (H5N1) was previously generated through reverse genetics with sequences based on Global Initiative on Sharing All Influenza Data (GISAID) accession EPI_ISL_19014384 with noncoding regions determined from consensus alignment of H5N1 strains from the 2.3.4.4b clade viruses (30).

### Fluorescence microscopy

rVSV plaque assays and infectivity assays were imaged for eGFP expression using an EVOS automated fluorescence microscope (Thermo Fisher) with a 4× objective. Images of whole wells were stitched together using integrated EVOS software and further processed using ImageJ software (National Institutes of Health).

### Monoclonal antibodies and IVIG

IgGs were produced as previously described (51) by transient transfection of heavy and light chain plasmids into 293 F cells using polyethylenimine (PEI) transfection reagent. Five days post-transfection, supernatants were collected, clarified by low-speed centrifugation, and incubated overnight with Protein A Agarose Resin (GoldBio) at 4°C. The resin was collected in a chromatography column and washed with one column volume of 10 mM tris(hydroxymethyl)aminomethane (tris), 150 mM NaCl at pH 7.5. IgGs were eluted in 0.1 M glycine (pH 2.5), which was immediately neutralized by 1 M Tris (pH 8.5). Antibodies were dialyzed against phosphate buffered saline (PBS) pH 7.4.

IVIG was procured from a commercial source (GAMMAGARD LIQUID, Takeda Pharmaceuticals) as a solution of 10% human immunoglobulin in 250 mM glycine. Immunoglobulin is at least 98% IgG, based on the manufacturer’s information.

### SDS-PAGE and Western blots

rVSVs were concentrated by ultracentrifugation over a 10% sucrose cushion and resuspended in PBS. Purified virus was boiled in Laemmli buffer under reducing conditions and run on a 4–20% acrylamide gel (BioRad). Gels were stained with Coomassie protein stain and imaged using a LICORbio Odyssey CLx imager (LICORbio). For Western blot analysis, gels were run as described, then transferred to nitrocellulose, blocked in 5% nonfat dry milk in PBS with 0.1% Tween 20 (PBST), and probed with mouse anti-VSV-M antibody 23H12 (0.1 µg/mL; Millipore Sigma) and anti-HA2 antibody LAH31 (29) (0.2 µg/mL) in 5% milk in PBST, followed by anti-mouse IR800 or anti-human IR800 (LICORbio) secondary antibodies. Membranes were imaged with a LiCOR Odyssey CLx imager.

### Neutralization assays

Microneutralization assays using A/dairy cattle/Texas/24008749001/2024 (H5N1) were performed as previously described (30). Briefly, twofold serial dilutions of monoclonal antibodies or IVIG were incubated with 10^3.3^ tissue culture infectious dose 50 (TCID_50_) of virus for one hour at room temperature with continuous rocking. Media were added to 96-well plates of confluent MDCK cells before the virus–antibody mixture was added. Cytopathic effect (CPE) was determined after four days, and neutralizing antibody titer was expressed as the reciprocal of the highest dilution of antibody required to completely neutralize infectivity. The concentration of antibody required to neutralize 100 TCID_50_ of virus was calculated based on the neutralizing titer dilution multiplied by the initial antibody concentration.

rVSV microneutralization assays were performed as above, with modification. Twofold serial dilutions of antibodies were incubated with 100 PFU of virus for one hour at room temperature with continuous rocking. Media were added to 96-well plates of confluent BSRT7 cells prior to the addition of the virus–antibody mixture. Infection was assessed by visual inspection for eGFP-positive infected cells after 1–2 days. Neutralizing concentration was determined as described above.

### Antibody escape

For antibody escape in bulk, 10^6^ PFU of rVSV-H5N1dc2024 was incubated with 10 µg/mL FLD194 IgG for one hour at room temperature with continuous rocking. Media were removed from one well of a 6-well plate of confluent BSRT7 cells and were replaced with virus–antibody mixture. Cells were incubated with the virus–antibody mixture at 34°C and monitored daily for eGFP expression until the virus had spread throughout the culture by four days. Viral supernatant was collected, clarified by low-speed centrifugation, and titer determined by plaque assay on BSRT7 cells. Viral genomic RNA was extracted and sequenced as described above.

For antibody escape by plaque selection, 6-well plates of confluent BSRT7 cells were infected with 10^6^ PFU of rVSV-H5N1dc2024 for one hour at 37°C. The virus was removed, and an agarose overlay containing 10 µg/mL FLD194 IgG or 65C6 IgG was added. Cells were incubated at 34°C and monitored for eGFP expression. At 3–5 days postinfection, large plaques were identified in each condition, picked, and grown on BSRT7 cells in the presence of 10 µg/mL of the respective antibody. Viral stocks were titered and sequenced as described above.

### Oseltamivir resistance assays

Oseltamivir (Millipore Sigma) was reconstituted in sterile water. Serial twofold dilutions of oseltamivir in DMEM were mixed with 100 PFU of rVSV-H5N1dc2024, rVSV-H5N1dc2024-NA-H275Y, or rVSV-eGFP. Media were removed from 96-well plates of confluent BSRT7 cells and replaced with oseltamivir–virus mixture. Cells were imaged two days postinfection, and the images were processed as described above.

## Data Availability

All data are reported here. No ancillary data sets were generated in this study. Data files are available upon request.
